# The ERAP1 active site cannot productively access the N-terminus of antigenic peptide precursors stably bound onto MHC class I

**DOI:** 10.1038/s41598-021-95786-x

**Published:** 2021-08-13

**Authors:** George Mavridis, Anastasia Mpakali, Jerome Zoidakis, Manousos Makridakis, Antonia Vlahou, Eleni Kaloumenou, Angeliki Ziotopoulou, Dimitris Georgiadis, Athanasios Papakyriakou, Efstratios Stratikos

**Affiliations:** 1 Protein Chemistry Laboratory, National Centre for Scientific Research Demokritos, 15341 Agia Paraskevi, Greece; 2 Laboratory of Organic Chemistry, Department of Chemistry, National and Kapodistrian University of Athens, 15784 Panepistimiopolis Zografou, Greece; 3 Centre of Basic Research, Biomedical Research Foundation of the Academy of Athens, 11527 Athens, Greece; 4 Biochemistry Laboratory, National and Kapodistrian University of Athens, 15784 Panepistimiopolis Zografou, Greece

**Keywords:** Enzyme mechanisms, Peptides, Proteases, Biochemistry, Biophysics, Immunology

## Abstract

Processing of N-terminally elongated antigenic peptide precursors by Endoplasmic Reticulum Aminopeptidase 1 (ERAP1) is a key step in antigen presentation and the adaptive immune response. Although ERAP1 can efficiently process long peptides in solution, it has been proposed that it can also process peptides bound onto Major Histocompatibility Complex I molecules (MHCI). In a previous study, we suggested that the occasionally observed “ontο MHCI” trimming by ERAP1 is likely due to fast peptide dissociation followed by solution trimming, rather than direct action of ERAP1 onto the MHCI complex. However, other groups have proposed that ERAP1 can trim peptides covalently bound onto MHCI, which would preclude peptide dissociation. To explore this interaction, we constructed disulfide-linked MHCI-peptide complexes using HLA-B*08 and a 12mer kinetically labile peptide, or a 16mer carrying a phosphinic transition-state analogue N-terminus with high-affinity for ERAP1. Kinetic and biochemical analyses suggested that while both peptides could access the ERAP1 active site when free in solution, they were unable to do so when tethered in the MHCI binding groove. Our results suggest that MHCI binding protects, rather than promotes, antigenic peptide precursor trimming by ERAP1 and thus solution trimming is the more likely model of antigenic peptide processing.

## Introduction

Major Histocompatibility Class I molecules (MHCI) bind small peptides (usually 8–10 amino acids long) originating from the proteolysis of intracellular proteins and the complex is translocated on the cell surface^1^. There, the complex can interact with specialized receptors located on the surface of cytotoxic T-lymphocytes, and this interaction can lead to the lysis of the target cell, if the peptides in the complex are recognized to belong to pathogens or are altered as a result of malignant transformation. These peptides (called antigenic peptides if they elicit T-cell responses) are generated inside the cell by a cascade of proteolytic events, the last of which includes the trimming of N-terminal residues of elongated precursors by ER-resident aminopeptidases^2^. ER aminopeptidase 1 (ERAP1) has been demonstrated to be crucial for the generation of many antigenic peptides and to be able to indirectly regulate adaptive immune responses by influencing the repertoire of antigenic peptides presented on the cell-surface, often referred to as the immunopeptidome^3^. ERAP1 is polymorphic and several common allotypes exist in the population affecting its functional properties and predisposition to disease such as cancer and autoimmunity^4,5^. Due to its important role in adaptive immune responses, ERAP1 is an emerging target for cancer immunotherapy and inflammatory diseases with autoimmune etiology^6–8^.

ERAP1 is a member of the oxytocinase subfamily of M1 aminopeptidase family^9^ and its ability to trim peptides in solution has been well documented^10,11^. Its specific role in trimming antigenic peptide precursors has been associated with some unusual molecular properties, such as preferences for length and specific recognition of the C-terminus of the peptide^12,13^. In addition to this more classical mechanism, it has been proposed that ERAP1 can also trim antigenic peptides while they are bound onto MHCI^14,15^. Deciphering which mechanism (in solution versus onto-MHCI) is more important for generating antigenic peptides is crucial, because each of the two mechanisms implies distinct molecular criteria for the selection of antigenic peptides in the cell^16^. If ERAP1 processes peptides in solution, then interactions between the peptide and ERAP1, such as the ones recently observed in ERAP1-peptides crystal structures^13^, should underlie the effects of ERAP1 on the immunopeptidome^3^. Conversely, if ERAP1 trims peptides while they are bound onto MHCI, then MHCI-peptide interactions should be the main driver. Knowing which mechanism is dominant is critical since it alters our perception on the criteria for antigenic peptide selection and our ability to predict antigenic epitopes or influence their production pharmacologically^16^.

In a recent paper from our group, we examined the kinetics of ERAP1-trimming of antigenic peptide precursors bound onto three different MHCI alleles and concluded that in all cases MHCI protected the peptides from ERAP1 trimming, thus suggesting that solution trimming is the main mechanism of ERAP1-mediated antigenic peptide generation^17^. We furthermore discovered that even in a single case of peptide-MHCI allele pair that onto-MHCI trimming was observed, this effect was not ERAP1-specific and likely proceeded via rapid dissociation of the peptide followed by solution trimming. While however, this mechanism could explain most of the observed effects in the literature, some published experiments include a peptide tethered by disulfide bond onto the MHCI binding groove^15,18^. Since tethering the peptide by a disulfide-bond would preclude rapid dissociation and solution trimming by ERAP1, we decided to examine this system further. For this, we engineered a disulfide linkage between HLA-B*08:01 and a 12mer kinetically labile peptide and examined its sensitivity to ERAP1. We furthermore engineered a disulfide-linked 16mer peptide carrying an N-terminus that is a transition-state analogue with high affinity for the ERAP1 catalytic center and examined its ability to access the ERAP1 active site. Results presented in this study clearly suggest that tethering the C-terminus of a peptide to the MHCI binding groove makes its N-terminus not readily accessible to ERAP1, thus making onto-MHCI trimming an unlikely event.

## Results

### Construction of a disulfide-linked HLA-B*08/peptide complex

**Figure 1:**
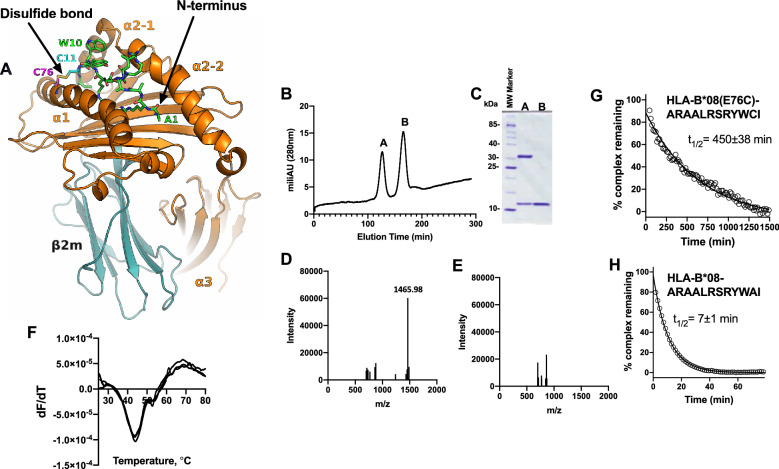
Construction and validation of a disulfide-linked HLA-B*08/peptide complex. Panel (**A**), molecular model of the HLA-B*08(E76C)-ARAALRSRYWCI complex (peptide shown in green sticks, disulfide bond between the HLA residue Cys76 and peptide residue Cys11, is indicated). Panel (**B**), size-exclusion chromatogram after refolding of HLA-B*08(E76C) with the peptide ARAALRSRYWCI. Panel (**C**), SDS-PAGE of peaks A and B of chromatogram shown in Panel (**B**). Panel (**D**), MALDI-TOF–MS of purified complex in the presence of 100 mM DTT. Panel (**E**), MALDI-TOF–MS of purified complex in the absence of DTT. Panel (**F**), 1st derivative of thermal shift assay of purified complex (three repetitions shown). Panel (**G**), kinetic analysis of binding of SYPRO Οrange to purified complex HLA-B*08(E76C)-ARAALRSRYWCI (each data point is the average of three repetitions). Panel (**H**) Kinetic analysis of binding of SYPRO Οrange to non-covalent complex HLA-B*08 – ARAALRSRYWAI (each data point is the average of two repetitions).

We have previously demonstrated that the 12mer peptide with the sequence ARAALRSRYWAI (an N-terminally extended version of the epitope ELRSRYWAI from nucleoprotein of influenza A virus^19^) can be refolded with HLA-B*08:01 to form a thermodynamically stable but kinetically labile complex and that ERAP1 can apparently trim this peptide while it is bound onto HLA-B*08:01, albeit slower than in solution^17^. To further explore whether it is necessary for the peptide to dissociate from HLA-B*08 before being trimmed by ERAP1, as suggested previously^17^, we constructed a disulfide-linked version of the complex using the E76C mutation in HLA-B*08 that introduces a cysteine residue in the binding groove of the MHCI^18^. This approach has been previously used successfully to trap peptides in the MHCI binding groove while retaining their structure and immunogenicity^20^. HLA-B*08(E76C) was refolded in the presence of a peptide with the sequence ARAALRSRYW**C**I which should juxtapose the cysteine residue in the peptide and the engineered cysteine residue in HLA-B*08(E76C) allowing for the formation of a disulfide bond that should covalently link the peptide and the HLA (Fig. 1A). Refolding heavy chain HLA-B*08(E76C) with beta-2 microglobulin and ARAALRSRYWCI was efficient and allowed the isolation of the complex at reasonable yield (Fig. 1B and C). The presence of the correct peptide bound onto HLA-B*08 was confirmed by MALDI-TOF–MS (Fig. 1D and E). The correct MS signal for the peptide’s MW was only detectable if the complex was treated with 100 mM DTT, which indicated that the desired disulfide bond was formed in the refolded complex.

To confirm the stability of the constructed HLA-B*08(E76C)-ARAALRSRYWCI complex, we first analyzed it using the Thermal Shift Assay^17^. The complex had a melting temperature of 44.0 ± 0.5 °C (Fig. 1F) which is similar to the melting temperature calculated for the non-disulfide-linked analogue HLA-B*08-ARAALRSRYWAI^17^, suggesting that introduction of the disulfide bond does not significantly contribute to the thermodynamic stability of the complex. The kinetic stability of the complex however was greatly increased. Using a competition assay with the SYPRO Orange dye^21^, we calculated that the half-life of the HLA-B*08(E76C)-ARAALRSRYWCI complex was 450 ± 38 min at 37 °C (Fig. 1G). In sharp contrast, the half-life of the non-covalent HLA-B*08-ARAALRSRYWAI complex was 7 ± 1 min (Fig. 1H), consistent with previous measurements^17^. These results suggest that, while the dye can still access the MHC binding groove to bind even when the peptide is covalently linked, covalently linking the peptide slows this process down by almost 2 orders of magnitude.

### Stability of B08-AI12Cys versus peptide exchange and ERAP1 trimming

**Figure 2:**
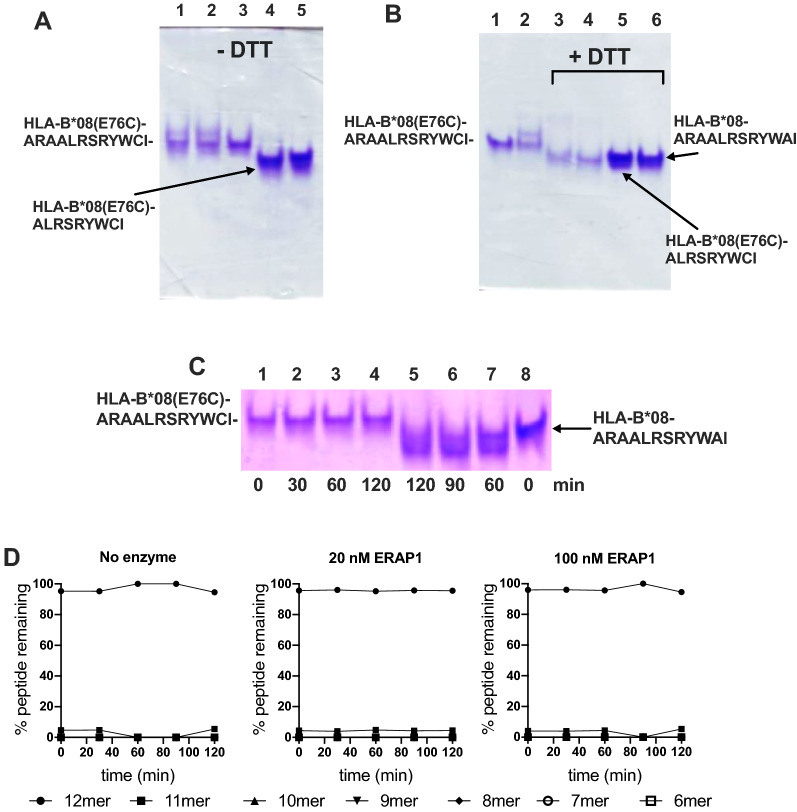
Stability of HLA-B*08(E76C)-ARAALRSRYWCI versus peptide exchange and ERAP1 trimming. Panel (**A**) Νative-PAGE of HLA-B*08(E76C)-ARAALRSRYWCI mixed with excess ALRSRYWAI peptide: *lane 1*, complex incubated for 60 min at RT; *lane 2*, complex incubated with 100 μΜ ALRSRYWAI peptide for 60 min at RT; *lane 3*, complex without incubation; *lane 4*, HLA-B*08(E76C)-ALRSRYWCI complex; *lane 5*, HLA-B*08-ARAALRSRYWAI complex. Panel (**B**) lane 1, HLA-B*08(E76C)-ARAALRSRYWCI complex incubated at RT for 60 min; *lane 2*, HLA-B*08(E76C)-ARAALRSRYWCI complex + 100 μΜ ALRSRYWAI peptide, 60 min incubation; *lane 3*, HLA-B*08(E76C)-ARAALRSRYWCI + 100 μΜ DTT, 60 min incubation; *lane 4*, HLA-B*08(E76C)-ARAALRSRYWCI + 100 μΜ DTT + 100 μΜ ALRSRYWAI peptide; *lane 5*, HLA-B*08(E76C)-ALRSRYWCI; lane 6, HLA-B*08-ARAALRSRYWAI. Panel (**C**) Native PAGE of HLA-B*08(E76C)-ARAALRSRYWCI (lanes 1–4) or HLA-B*08-ARAALRSRYWAI (lanes 5–8) complexes incubated for 0–120 min with 20 nM ERAP1 (full-length gel is presented in Supplementary Fig. 1). Panel (**D**) MALDI-TOF–MS analysis of the HLA-B*08(E76C)-ARAALRSRYWCI complex incubated with ERAP1.

Covalently linking the ARAALRSRYWCI peptide would be expected to preclude peptide exchange. Indeed, incubating the HLA-B*08(E76C)-ARAALRSRYWCI complex with an excess of the high-affinity 9mer peptide with the sequence ALRSRYWAI (AI9) did not alter the electrophoretic mobility of the complex in native-PAGE suggesting no peptide exchange (Fig. 2A). In contrast, inclusion of DTT during the incubation with AI9 resulted in full exchange of the ARAALRSRYWCI with ALRSRYWAI (Fig. 2B), similarly to the non-covalently linked AI12 peptide shown in our previous publication^17^. Therein, we had also demonstrated that the non-disulfide linked AI12 peptide refolded with HLA-B*08, could be apparently trimmed by ERAP1 with a half-life of 18 min and that this reaction could be followed by native-PAGE^17^. To test whether the disulfide-linked peptide ARAALRSRYWCI would behave differently, we incubated the HLA-B*08(E76C)-ARAALRSRYWCI complex with 20 nM ERAP1 (Fig. 2C). For up to 2 h of reaction, no change in electrophoretic mobility was observed (Fig. 2C, lanes 1–4), suggesting that no significant trimming of the bound peptide was performed. In contrast, ERAP1 was able to modify the electrophoretic mobility of the non-covalently linked ARAALRSRYWAI peptide, as described previously (see reference^17^ and Fig. 2C, lanes 5–8). We conclude that covalently linking the peptide to the MHC binding groove is sufficient to protect its N-terminus from being trimmed by ERAP1.

To confirm the above finding we followed the trimming reaction by MALDI-TOF–MS which allows the concurrent detection of several peptide species. HLA-B*08(E76C)- ARAALRSRYWCI was incubated with either 20 nM or 100 nM of ERAP1 and the reaction was stopped by the addition of TFA and flash-frozen. MALDI-TOF–MS analysis was performed as described before^17^, but with the addition of 100 mM DTT to reduce the disulfide linkage to the complex so that the freed peptide can be detected (Fig. 2D). We observed no time-dependent decrease of the 12mer peptide, a stark difference from a similar analysis using the non-disulfide linked analogue peptide ARAALRSRYWAI published before, which was trimmed with a half-life of 20–30 min^17^. This result was consistent with the native-PAGE analysis and suggests that covalently tethering the C-terminal moiety of the peptide in the MHC binding groove is sufficient to fully protect its N-terminus from ERAP1 trimming.

### Construction of a disulfide-linked HLA-B*08/ phosphinic peptide complex

**Figure 3:**
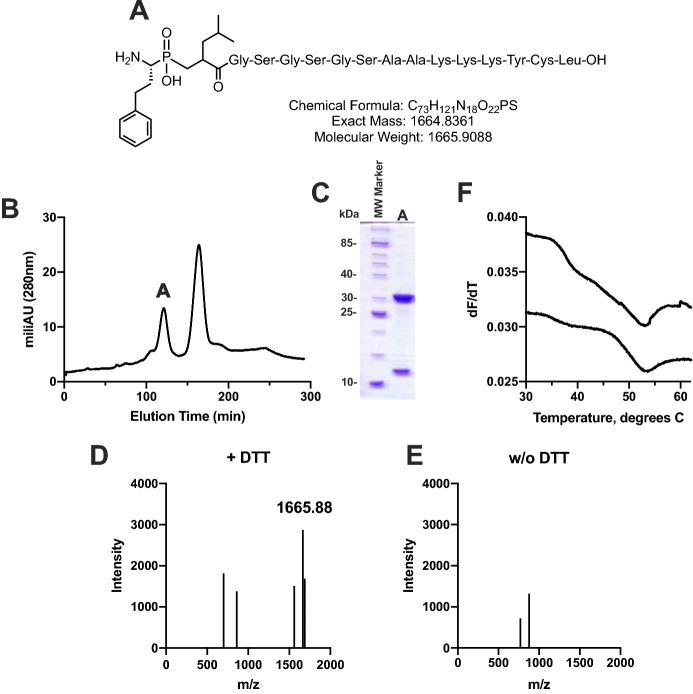
Construction of a disulfide-linked HLA-B*08/ phosphinic peptide complex. Panel (**A**) chemical structure of phosphinic pseudopeptide DG080. Panel (**B**) size-exclusion chromatogram of the refolding mixture of HLA-B*08(E76C) with DG080. Panel (**C**) SDS-PAGE analysis of peak A from the size-exclusion chromatogram shown in panel (**B**). Panel (**D**) MALDI-TOF–MS analysis of purified complex in the presence of 100 mM DTT. Panel (**E**) MALDI-TOF–MS analysis of purified complex in the absence of DTT. Panel (**F**) 1st derivative of thermal shift assay of purified complex (two representative experiments shown).

Phosphinic pseudopeptides are well-characterized transition-state analogues of M1 aminopeptidases and have been shown to bind to the active site of ERAP1 with high affinity^22^. To explore the potential interactions of the N-terminus of a peptide bound onto MHC and ERAP1, we designed a 16mer phosphinic pseudopeptide of the sequence hF*Ψ*[P(O)(OH)CH_2_]LGSGSGSAAKKKYCL (henceforth named peptide DG080, Fig. 3A). The peptide sequence is based on the HLA-B*08 restricted, HIV-1 Gag immunodominant epitope GGKKKYKL^23^, carrying a flexible N-terminal extension to allow it to reach into the ERAP1 active site and a cysteine at the penultimate C-terminal position. A similar N-extended peptide has been shown by x-ray crystallography to assume a conformation when bound onto HLA-B*08:01 so that its N-terminus extends away from the MHCI and we thus hypothesized that it would be appropriate for interacting with the ERAP1 active site^18^. This design should allow us to covalently tether the peptide onto HLA-B*08(E76C) and then explore whether the phosphinic N-terminus can access and bind to the active site of ERAP1. Refolding HLA-B*08(E76C) with DG080 was successful, albeit at low yields (Figs. 3B and C). The presence of the DG080 peptide in the MHC complex was validated by MALDI-TOF-MS of the purified complex (Fig. 3D and E). The correct molecular weight was only detectable in the presence of DTT, suggesting that the peptide was disulfide linked onto the MHC binding groove, as designed. Thermal Shift assay analysis revealed a melting temperature of 53.5 ± 0.5 °C which is in accordance with other HLA-B*08/peptide complexes investigated before^17,21^.

### DG080 fails to access the ERAP1 active site when disulfide-linked onto HLA-B*08

**Figure 4:**
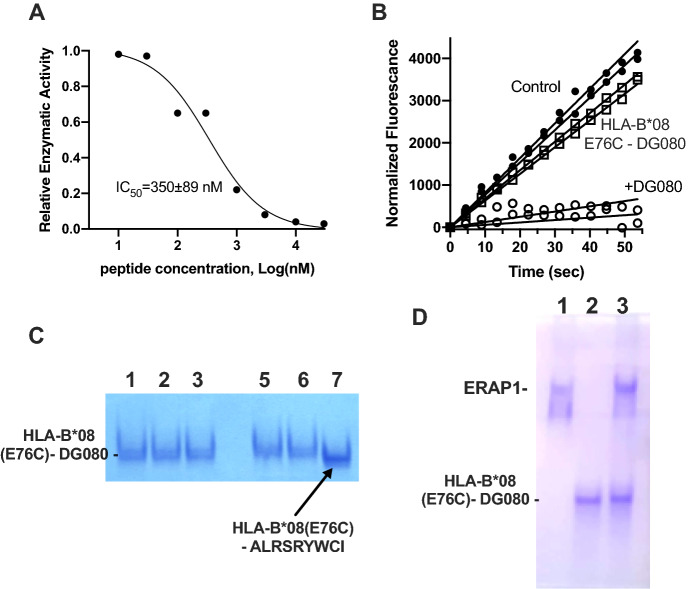
Characterization of the interaction between DG080 and the active site of ERAP1. Panel (**A**) effect of titration of free DG080 peptide on ERAP1 enzymatic activity (each point is the average of two measurements). Panel (**B**) time-dependent hydrolysis of fluorigenic ERAP1 substrate in the presence of 1 μΜ of either free DG080 or HLA-B*08 (E76C)-DG080 complex (two measurements per condition are shown). Panel (**C**) native-PAGE analysis of the stability of HLA-B*08(E76C)-DG080 complex: *lane 1*, complex incubated at 4 °C for 1 h; *lane 2*, complex incubated at 37 °C for 1 h; *lane 3*, complex incubated at 37 °C for 1 h in the presence of 20 nM ERAP1; *lane 5*, complex incubated at 37 °C for 1 h; *lane 6*, complex incubated at 37 °C for 1 h in the presence of 100 μM of peptide ALRSRYWAI; *lane 7*, HLA-B*08 (E76C)-ALRSRYWCI complex (full-length gel is presented in Supplementary Fig. 2). Panel (**D**) Native-PAGE of ERAP1 and HLA-B*08 (E76C)-DG080 complex: *lane 1*, ERAP1; *lane 2*, HLA-B*08 (E76C)-DG080 complex; *lane 3*, equimolar mixture of ERAP1 and HLA-B*08 (E76C)-DG080.

The phosphinic head of the DG080 pseudopeptide should provide it with high affinity for the active site of ERAP1. To validate this assumption, we mixed free DG080 with ERAP1 and followed ERAP1 activity using the small fluorigenic substrate L-Leucine-7-amido-4-methylcoumarin (Leu-AMC)^24^. The peptide inhibited ERAP1 activity in a dose-dependent manner with an IC_50_ of 350 ± 89 nM, confirming the high affinity of the phosphinic moiety for ERAP1’s active site (Fig. 4A). Given this result, we tested the ability of the HLA-B*08(E76C)-DG080 complex to inhibit ERAP1. Due to the low amounts of complex available, we performed assays at a fixed concentration of 1 μΜ for both free DG080 and HLA-B*08(E76C)-DG080 complex. At that concentration, free DG080 was able to inhibit almost 90% of ERAP1 activity, consistent with its measured IC_50_. In contrast, the HLA-B*08(E76C)-DG080 complex was unable to fully inactivate ERAP1, and had only a marginal effect on its enzymatic activity (Fig. 4B). We conclude that although DG080 in solution has full access to the ERAP1 active site, DG080 covalently bound onto HLA-B*08 has very limited access to the active site of ERAP1, possibly due to steric hindrances that limit a proper binding orientation necessary so that the ERAP1 active site can approach the MHCI binding groove^25^. To validate the stability of the HLA-B*08(E76C)-DG080 versus ERAP1 trimming or peptide exchange, we first incubated the complex with 20 nM ERAP1 at 37 °C for 1 h and analyzed the sample by native-PAGE (Fig. 4C). No change in electrophoretic mobility was observed. We furthermore, incubated the complex with an excess (100 μM) of the 9mer peptide ALRSRYWAI at 37 °C for 1 h (Fig. 4C). Again, no change in electrophoretic mobility was evident, suggesting that the DG080 peptide is stably bound onto HLA-B*08. To further explore a potential interaction between ERAP1 and the phosphinic N-terminus of the peptide bound onto HLA-B*08, we analyzed an equimolar mixture of ERAP1 with HLA-B*08(E76C)-DG080 by native PAGE (Fig. 4D). ERAP1 and HLA-B*08(E76C)-DG080 migrate as distinct bands in native PAGE, thus it should be possible to detect a stable molecular interaction by observing shifts on the gel. No band shifts were evident suggesting that a stable ERAP1-HLA-B*08(E76C)-DG080 ternary complex could not be formed. We conclude that although DG080 can bind and inhibit ERAP1 with high affinity, it cannot access the active site when tethered to the MHCI binding groove, possibly due to steric limitations.

## Discussion

In our previous work we had suggested that the occasional apparent trimming of antigenic peptide precursors bound onto MHCI alleles is rather a multi-step process that involves rapid peptide dissociation, solution trimming by ERAP1 and peptide re-binding^17^. This mechanism cannot, however, readily explain the observation of “onto MHCI” trimming in cases that the peptide is covalently linked in the MHCI binding grove as described in papers by James et al. and Bouvier et al.^15,18^, since the disulfide bond should not allow rapid peptide dissociation. To further examine this mechanism, we tethered a kinetically labile 12mer peptide onto the MHC, through a disulfide bond and investigated its trimming by ERAP1. A similar peptide, when untethered, was shown in our previous paper to rapidly dissociate from MHCI and to be efficiently trimmed by ERAP1, albeit slower than in solution^17^. In contrast, we find that the disulfide-tethered peptide is completely resistant to ERAP1 trimming. Furthermore, analysis of a similar complex consisting of a 16mer peptide tethered onto MHCI by its penultimate C-terminal residue and carrying a phosphinic group at its N-terminus with high-affinity for the ERAP1 active site, also suggested lack of productive interaction. Thus, in our experiments we find no evidence to support a productive enzymatic interaction between the active site of ERAP1 and a covalently-tethered onto the MHCI antigenic peptide precursor.

**Figure 5:**
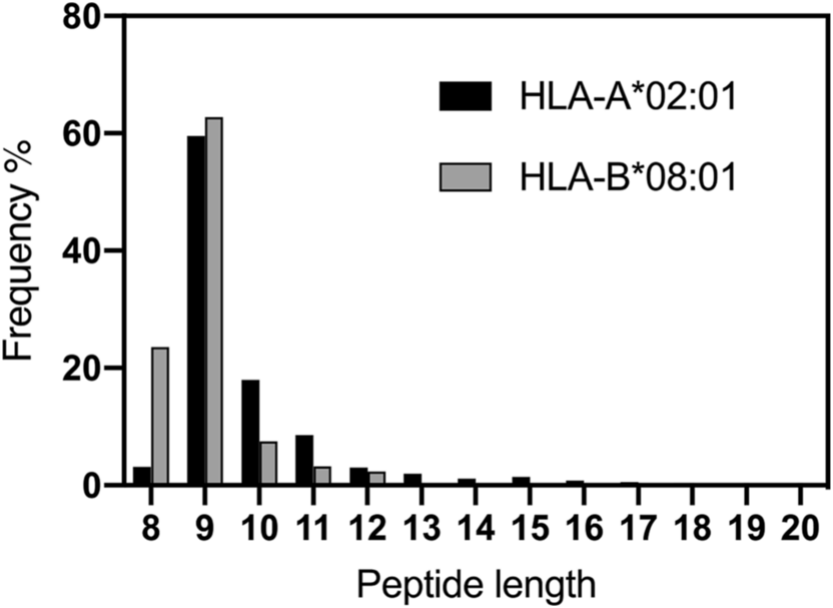
Length distribution of antigenic peptides that bind to HLA-B*08:01 and HLA-A*02:01. Data were extracted from the immune epitope database and analysis resource (www.iedb.org)^28^, 7231 total peptides for HLA-B*08:01 and 33,362 total peptides for HLA-A*02:01. Frequency distribution analysis was performed with Graphpad Prism 8.0™.

Although our results may be at first interpreted to constitute a discrepancy with previously published data by the James et al. and Bouvier et al.^15,18^, several possibilities exist that could reconciliate both sets of results. These are analyzed below:

In regards to the results in Li et al.^18^, the authors report ERAP1 trimming of a 20mer peptide but demonstrate no trimming products at or below 14 residues long. Furthermore, trimming of the 20mer peptide required a very large amount of enzyme (100-fold higher than in solution), suggesting a very low specific activity, consistent with unfavorable steric interactions between ERAP1 and the MHCI. In this context, our results are not really contradictory, since the cleavable peptide we utilized is 12 residues long. There is no mechanistic obstacle for an aminopeptidase to trim an elongated peptide bound onto a protein, provided it is sufficiently long to be able to reach inside the enzyme’s active site and steric clashes between the enzyme and the protein are avoided. Given the size of the ERAP1 internal cavity, peptides of 16 residues or longer may be able to achieve this^13,26^. This effect, however, is unlikely to be physiologically relevant, since most peptides that enter the ER through the action of the Transporter Associated with Antigen Processing (TAP) are 16 amino acids or shorter^27^, and antigenic peptides are rarely over 11 amino acids (97% of known antigenic peptides that bind HLA-B*08:01 are 11 residues or shorter, Fig. 5).

In regards to the results described by E. James and co-workers^15,29^, it should be noted that the experiments were performed within a cellular context and using an indirect measurement of ERAP1 activity (T-cell activation), which lack the ability for kinetic analysis and thus a direct comparison between different trimming mechanisms. A possible discrepancy between in vitro kinetics and cell-based behavior should be interpreted carefully and at the same time can constitute an opportunity because it may reveal additional molecular components that are missing from the in vitro setup but which are crucial for our understanding of the biochemical pathway. For example, it is possible that an adaptor protein could enhance ERAP1 interaction with the MHCI, although it would have to be an unknown component since our efforts to detect interactions with the Peptide Loading Complex did not yield positive results^17^. Another possibility is that the high concentrations achievable in local ER microenvironments could promote a weak protein–protein interaction that is difficult to reproduce in vitro. Alternatively, ERAP1 may recognize particular MHCI transient conformations that are not present upon in vitro refolding. It is however also possible that the redox state of the ER that promotes disulfide shuffling^30^ could result in partial reduction of the disulfide bond holding the peptide onto the MHCI and subsequent rapid dissociation that is followed by ERAP1 trimming and rapid re-binding. That possibility could resolve the apparent contradiction with our results. Finally, it should be noted that the recent discovery of a small MW inhibitor of ERAP1 that targets the enzyme’s regulatory site that normally binds the peptidic substrate’s C-terminus, clearly suggests that the antigenic peptide’s C-terminus needs to be available to interact with ERAP1 and therefore not available to interact with the MHCI^31^. This inhibitor was fully active in a cellular assay of antigen presentation, suggesting that the interaction between ERAP1 and the C-terminus of antigenic peptides is necessary inside the cell and it is thus unlikely that ERAP1 can trim peptides that have their C-terminus sequestered inside the MHCI binding groove.

A mechanism of “onto-MHC” trimming for ERAP1 implies a possible direct molecular interaction between MHCI and ERAP1. However, no such direct interaction between ERAP1 and MHCI has been demonstrated so far. While such an interaction is conceptually attractive, in the context of constituting an active link between antigen processing and antigen presentation, it is by no means necessary and experimental observations can be largely explained by solution trimming and the dynamic nature of MHCI-peptide interactions. We propose that until such an interaction is unequivocally established, the simpler model of solution trimming is the most consistent with what we understand about the mechanism of action of this enzyme and the mechanism of peptide loading onto MHCI and should therefore be the preferred model.

## Methods

### Peptides

Peptides ARALARSRYWAI, ARALARSRYWCI ALRSRYWAI and ALRSRYWCI were synthesized by GeneCust, France. All peptides were purified by HPLC using a reversed-phase C18 column (Merck, USA) and eluted using an acetonitrile gradient. Peptide purification was validated by mass spectrometry. All peptides used for refolding were > 90% pure.

### Site directed mutagenesis

Mutagenesis in order to introduce the E76C mutation to HLA B*08:01 heavy chain was performed by using the Quikchange II kit (Agilent technologies) following the manufacturer’s instructions. The primers sequences used were the following: forward, 5′-gttccgcaggctgcatcggtcagtctgtgtgttggtct-3′; reverse, 5′-agaccaacacacagactgaccgatgcagcctgcggaac-3′.

### HLA-B*08, HLA-B*08:01 (E76C) and b_2_m expression and purification

Both HLA-B*08:01 heavy chain, HLA-B*08:01 (E76C) heavy chain and beta-2 microglobulin were expressed as inclusion bodies in *Escherichia coli* strain BL21 DE3 cells as reported previously^17^.

### Formation of MHC I complexes

Folding in vitro of the complexes B*08:01(E76C)-ARALARSRYWCI, ΗLA-B*08:01(E76C)-DG080, HLA-B*08:01-ARALARSRYWAI and HLA-B*08(E76C)-ALRSRYWCI was performed as previously reported^17^. Briefly, urea-solubilized inclusion bodies of heavy chain and β_2_-microglobulin were mixed in the presence of excess concentration of peptide and dialyzed against folding buffer (0.4 M arginine (AppliChem, A3675), 100 mM Tris–HCl, pH 8, 2 mM EDTA, 5% (v/v) glycerol, 5 mM reduced GSH (AppliChem, A2084), 0.5 mM L-GSH oxidized (Acros Organics, 320,220,050), and 0.1 mM PMSF (AppliChem, A0999). After dialysis the mixture was purified by size-exclusion chromatography (Sephacryl S-200) and the peak corresponding to refolded MHCI was concentrated by ultrafiltration and stored at − 80 °C in the presence of 10% glycerol (v/v).

### Expression and purification of ERAP1

Expression and purification of ERAP1 was performed as described previously^24^. Briefly, ERAP1 was expressed in Hi5 insect cells after infection with recombinant baculovirus at 27 °C. Protein was isolated by affinity chromatography using nickel-nitrilotriacetic acid–agarose beads. Protein aliquots were kept at − 80 °C in Hepes/NaCl buffer pH 7.0 supplemented with 10% (v/v) glycerol, until needed.

### Fluorigenic enzymatic assay

ERAP1 enzymatic activity and inhibitor IC_50_ values were calculated as previously reported^24^. Briefly, hydrolysis of the fluorescent substrate leucine-aminomethylcoumarin (Leu-AMC, Sigma–Aldrich, L2145) was followed at 460 nm (excitation at 380 nm) using a Spark 10 M (TECAN) multimode microplate reader. The IC_50_ of DG080 was calculated by using the equation log (inhibitor) versus response-variable slope of GraphPad Prism 8.0™.

### MALDI-TOF-mass Spectrometry

Detection of peptides bound onto ΗLA-B*08:01 by MALDI-TOF–MS was performed as previously described^17^ with the exception that 100 mM DTT was added to 10 μL samples of ΗLA-B*08:01(E76C) protein complexes to break the disulfide bond and allow detection of the peptide.

### Differential scanning fluorimetry assay (DSF)

The assay as well as the calculation of the melting temperature (*T*_*m*_) of the complexes HLA-B*08:01(E76C)-DG080 and HLA-B*08:01(E76C)-AI12Cys were performed as previously reported^17^.

### Peptide dissociation followed by SYPRO-ORANGE

The assay was performed in the Lightcycler 96 RT-PCR instrument as previously reported^17^. Reaction mixtures (total volume 20 μL) consisted of 16 μL protein complex (final concentration 8 μΜ) and 4 μL of 50X SYPRO Orange dye (Sigma–Aldrich, S5692). Excitation wavelength was set at 533 nm and emission at 572 nm. The temperature was set at 37 °C for the duration of the experiment.

### Polyacrylamide gel electrophoresis under non-denaturing conditions (Native-PAGE)

Separating gel was prepared by mixing 2.9 mL Tris–HCl 0.375 M pH 8.8 solution, 2 mL acrylamide solution 30%, 100 μL APS 10% w/v and 5 μL TEMED. Stacking gel was formed by mixing 2.14 mL Tris–HCl 0.375 M pH 8.8 solution, 0.34 mL acrylamide solution 30%, 50 μL APS 10% w/v and 5 μL TEMED. Sample loading buffer was formed by mixing 62.5 mM Tris–HCl pH 6.8 solution, 25% v/v glycerol and 1% v/v bromophenol blue solution. Running buffer consists of 25 mM Tris-base and 192 mM glycine. Electrophoresis is conducted at 4 °C, 100 V for 5 h. 1,5 µg of MHC I-peptide complexes and 3 µg of ERAP1 were used.

### Synthesis of DG080

The preparation of phosphinic pseudopeptide DG080 {H-hFΨ[P(O)(OH)CH_2_]LGSGSGSAAKKKYCL-OH} was performed on solid phase by application of Fmoc protocol and using pin technology. Lanterns bearing the trityl alcohol linker (15 µmol/pin) were treated with a 1:10 v/v mixture of freshly distilled acetyl chloride/dry dichloromethane for 3 h at room temperature. The resulting trityl chloride lanterns were washed and subsequently added to a solution of Fmoc-Leu-OH (30 µmol/pin) and *N*,*N*-diisopropylethylamine (18 µL/pin) in dry dichloromethane (0.4 mL/pin) at room temperature. After 24 h, calculation of resin loading was realized by acidic cleavage (rt, 1 h) of a pre-weighed lantern with 0.5% trifluoroacetic acid/dichloromethane and it was found > 12 μmol/pin. Two loaded pins were subjected to standard Fmoc deprotection by applying 3 repetitions of treatment with a solution of 20% piperidine in *N*,*N*-dimethylformamide (0.5 mL/pin, 20 min for each repetition). After washing the lanterns with *N*,*N*-dimethylformamide (5 × 0.5 mL/pin) and dry dichloromethane (3 × 0.5 mL/pin), they were added to a solution of Fmoc-Cys(Trt)-OH (45 μmol/pin), diisopropylcarbodiimide (DIC, 45 μmol/pin) and 1-hydroxybenzotriazole (HOBt, 45 μmol/pin) in dichloromethane/DMF (6/1) (0.4 ml/pin). The resulting mixture was agitated for 4–5 h at room temperature and completion of coupling reaction was confirmed by Kaiser test. The above deprotection/coupling procedure was repeated for the next 12 natural aminoacids. All amino acids bearing functional groups at their side-chain were trityl (Trt)-protected except Lys which was Βοc-protected. After the addition of the first 14 aminoacids, Fmoc deprotection followed and the resulting pins were treated with a solution of Boc-(R)hPheΨ[P(O)(OAd)CH2](R,S)LeuOH (20 µmol/pin)^32^, diisopropylcarbodiimide (DIC, 20 μmol/pin) and 1-hydroxybenzotriazole (HOBt, 20 μmol/pin) in dichloromethane/DMF (6/1) (0.4 ml/pin) for 24 h at room temperature. Two repetitions of the coupling step were necessary for completion of the reaction. DG080 was obtained after deprotection and final detachment from the solid support using a solution of trifluoroacetic acid/thioanisole/1,2-ethanedithiol/amisole (90/5/3/2). After 2 h of agitation at room temperature, the crude peptide was isolated after precipitation with dry diethyl ether at 0 °C and RP-HPLC purification. [ESMS m/z (z = 2): calculated for [C_73_H_121_Ν_18_Ο_22_PS + 2H]^+^ 832.9; found: 833.4].

## Supplementary Information

Supplementary Information.

## Data Availability

All data described are available in the article and associated supporting information. Numerical values used for generation of graphs are available upon request to the corresponding author (Efstratios Stratikos; E-mail: stratos@rrp.demokritos.gr or estratikos@chem.uoa.gr).
