# Supplementary material for: The ERAP1 active site cannot productively access the N-terminus of antigenic peptide precursors stably bound onto MHC class I
Source: Sci Rep. 2021 Aug 13;11:16475. doi: 10.1038/s41598-021-95786-x (PMC8363620; doi:10.1038/s41598-021-95786-x)
Supplement: Supplementary file 1 — Supplementary Information. [file 41598_2021_95786_MOESM1_ESM.docx]

**Supplemental Figures**

**The ERAP1 active site cannot productively access the N-terminus of antigenic peptide precursors stably bound onto MHC class I**

George Mavridis^1^, Anastasia Mpakali^1^, Jerome Zoidakis^3^, Manousos Makridakis^3^, Antonia Vlahou^3^, Eleni Kaloumenou^2^, Angeliki Ziotopoulou^2^, Dimitris Georgiadis^2^, Athanasios Papakyriakou^1^, and Efstratios Stratikos^1,4,*^

^1^Protein Chemistry Laboratory, National Centre for Scientific Research Demokritos, Agia Paraskevi 15341, Greece

^2^Laboratory of Organic Chemistry, Department of Chemistry, National and Kapodistrian University of Athens, Panepistimiopolis Zografou 15784, Greece

^3^Centre of Basic Research, Biomedical Research Foundation of the Academy of Athens, Athens 11527, Greece

^4^Biochemistry Laboratory, National and Kapodistrian University of Athens, Panepistimiopolis Zografou 15784, Greece

^*^To whom correspondence should be addressed: Biochemistry Laboratory, National and Kapodistrian University of Athens, Panepistimiopolis Zografou 15784, Greece, E-mail: [estratikos@chem.uoa.gr](mailto:estratikos@chem.uoa.gr) or [stratos@rrp.demokritos.gr](mailto:stratos@rrp.demokritos.gr).


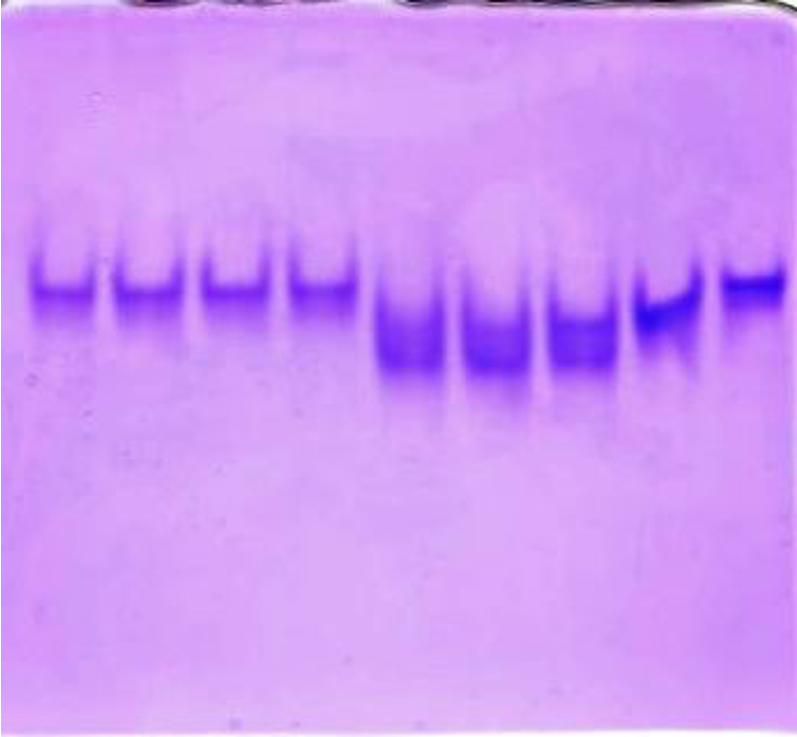


**Supplemental Figure 1**: Uncropped photograph of gel presented in Figure 2C


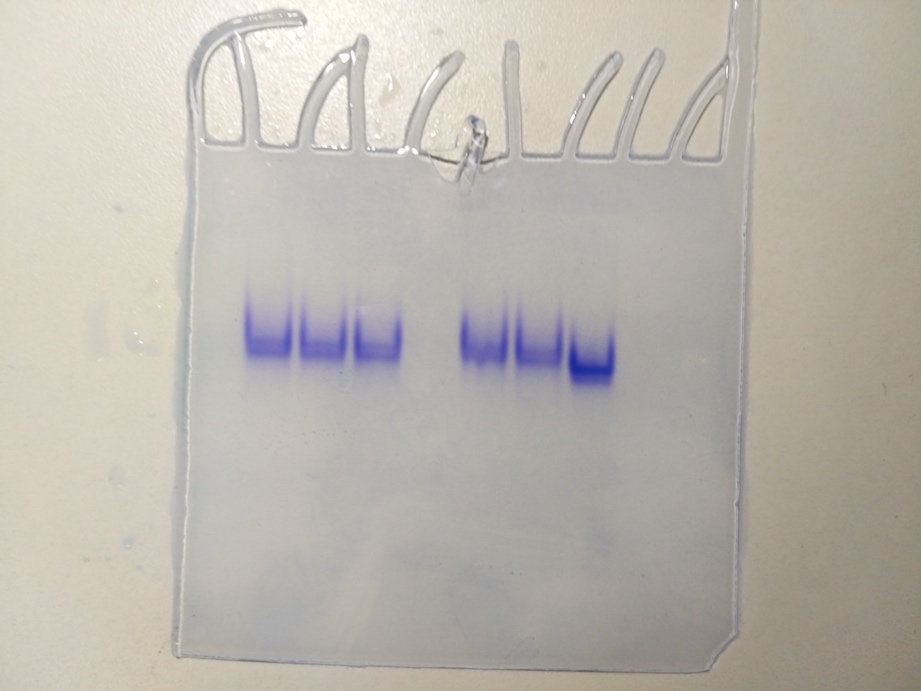


**Supplemental Figure 2:** Uncropped photograph of gel presented in Figure 4C
